# Products of sugar beet processing as raw materials for chemicals and biodegradable polymers

**DOI:** 10.1039/c7ra12782k

**Published:** 2018-01-17

**Authors:** J. Tomaszewska, D. Bieliński, M. Binczarski, J. Berlowska, P. Dziugan, J. Piotrowski, A. Stanishevsky, I. A. Witońska

**Affiliations:** Institute of General and Ecological Chemistry, Lodz University of Technology 116 Zeromskiego Street Lodz 90-924 Poland izabela.witonska@p.lodz.pl +48 42 631 30 94; Institute of Polymer & Dye Technology, Lodz University of Technology 12/16 Stefanowskiego Street Lodz 90-924 Poland; Institute of Fermentation Technology and Microbiology, Lodz University of Technology 171/173 Wolczanska Street Lodz 90-924 Poland; National Sugar Company S.A. 12 John Paul II Avenue Warsaw 00-001 Poland; Department of Physics, University of Alabama at Birmingham Birmingham AL 35294 USA

## Abstract

This paper presents an overview of alternative uses for products of sugar beet processing, especially sucrose, as chemical raw materials for the production of biodegradable polymers. Traditionally, sucrose has not been considered as a chemical raw material, because of its use in the food industry and high sugar prices. Beet pulp and beetroot leaves have also not been considered as raw materials for chemical production processes until recently. However, current changes in the European sugar market could lead to falling demand and overproduction of sucrose. Increases in the production of white sugar will also increase the production of waste biomass, as a result of the processing of larger quantities of sugar beet. This creates an opportunity for the development of new chemical technologies based on the use of products of sugar beet processing as raw materials. Promising methods for producing functionalized materials include the acidic hydrolysis of sugars (sucrose, biomass polysaccharides), the catalytic dehydration of monosaccharides to HMF followed by catalytic oxidation of HMF to FDCA and polymerization to biodegradable polymers. The technologies reviewed in this article will be of interest both to industry and science.

## Abstract 2

This paper presents an overview of alternative uses for products of sugar beet processing, especially sucrose, as chemical raw materials for the production of biodegradable polymers.

## Graphical abstract

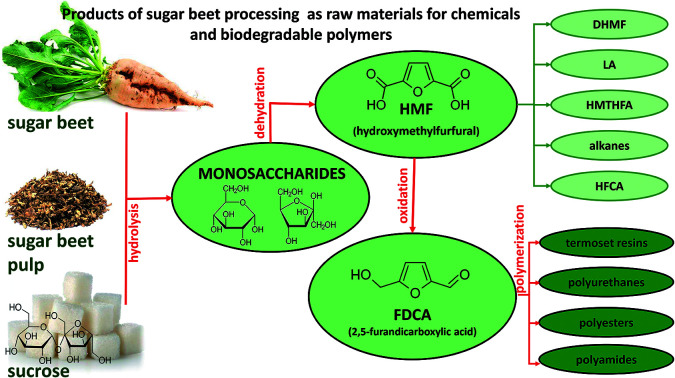

## Introduction

Most sugar production in Europe is from sugar beets. Both the cultivation of sugar beets and sugar production processes are subject to strict regulation by the Common Market Organisation, including quotas, minimum sugar prices and import volumes. This unique legal regulatory system was introduced in 2006,^1^ with changes beginning in 2013. In autumn 2017, the current production quotas, which guarantee minimum prices for sugar, will be abolished. This will cause dramatic changes in the European sugar market, in particular regarding the competitiveness of EU producers. These changes are intended to end the predominance of the largest manufacturing companies, which have plants in several EU Member States simultaneously, and to allow even small entrepreneurs to survive in the market.^2^

Consumption of sugar in the EU is rising steadily, mainly as a result of increasing immigration and the growing population of Europe. However, sugar beet growers and sugar producers face ever greater difficulties. Market projections for the year 2016/2017 indicate that sugar consumption in the EU will reach the maximum ceiling, largely as an effect of health-education campaigns and activities aimed at reducing the amount of sugar in the diet.^3^ Another problem facing European sugar producers is the influx of cheap cane sugar. Sweeteners are also likely to become more competitive.^4^ Food manufacturers will probably use more high-fructose corn syrup (HFCS), which has several advantages over conventional sugar, in terms of taste, stability, freshness and consistency. The potential effects of these trends can already be observed in the USA, where HFCS is the predominant sweetener used in beverages, sauces and other food products.^5^ Finally, with the loosening of EU restrictions, the export of white sugar will no longer be subject to tight limitations. The most competitive companies therefore intend to increase export production and look for new markets. However, these actions will have very low profit margins, and growth will be achieved by optimizing processes, rather than through additional investment. Less competitive manufacturers are likely to be eliminated or absorbed by the more powerful companies.

The market is thus becoming increasingly difficult for producers of white sugar derived from sugar beet. An alternative use of white sugar is for the production of bioethanol. However, in 2015 the competitive market conditions made the European bioethanol industry based on white sugar and sugar beet juice fermentation economically unprofitable. Reasons for this included a reduction in petroleum prices and the falling price of cereals, from which bioethanol is also produced. It is estimated that, unless petroleum prices rise significantly, levels of bioethanol production from white sugar or sugar beet juice will at best remain stable.^4^

Producing larger quantities of white sugar will also result in the production of more bio-waste from technological processes. This requires the development of new technologies for using waste from sugar factories, in addition to uses as feed or green manure in agriculture.

In view of the current and projected changes in the sugar market, producers are looking at developing alternative business models. This is a task not only for sales and marketing specialists, but also for chemists, bio-technologists and innovators, who may be able to find unconventional applications for sucrose. For economic reasons, sucrose has never been considered as a chemical raw material. However, in the context of falling prices and surplus capacity in the sugar industry, sucrose could be used in the production of valuable chemical compounds, such as biodegradable polymers.

## By-products obtained from the sucrose manufacturing processes

The fact that beet roots contain sugar was discovered in 1705 by Oliver de Serres, the famous French agronomist. However, the discovery was not acted upon. Half a century later, in 1747, the German chemist Andreas Marggraf demonstrated that the sugar crystals formed in a water solution of sugar beet juice were identical with sugarcane crystals. His student, Karl Achard, then developed an industrial process for extracting sugar from beets. This was the beginning of the sugar production from sugar beets in Europe.^6^

**Fig. 1:**
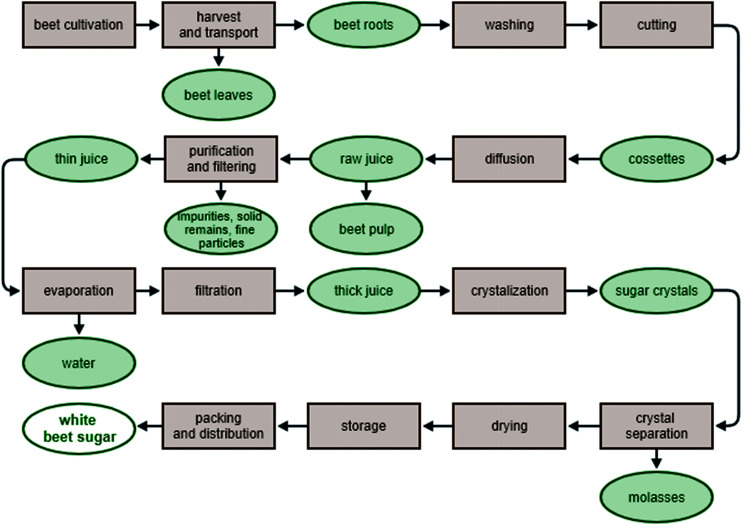
Simplified scheme for the production of sugar from sugar beet.

Sucrose is extracted from sugar beet using hot water. This results in raw juice, which is then purified, filtered and concentrated by cyclic rinsing and evaporation. To obtain the final product, the thick juice is crystallized. The resulting white sugar is then recrystallized, which ultimately leads to the production of high quality refined sugar (Fig. 1). Various sugar beet products are produced at different stages of beet processing. The by-product, which contains a large amount of water, comprises up to 75% of the beet pulp. This is used as a heat source and, circulating in a closed system, can be used repeatedly to provide a large proportion of the heat demands of a sugar production line. Following the extraction of sucrose, the sugar beet pulp and beet splinters are used primarily in animal feed or biogas production. Attempts are also being made to use beet leaves in the production of methanol.^7^ After the centrifugation of the thick syrup (the final process of sugar production), the molasses obtained are used mainly for the production of alcohol, in animal feed, or as a medium for yeast biomass production.^8^

Sugar products can be processed in a variety of ways, to produce not only sugar for food or feed additives but also valuable chemicals that can be used in biofuels, synthetic materials and pharmaceuticals.^9^ For example, raw sugar beet juice is considered an attractive feedstock for ethanol fermentation, due to its high fermentable sugar content. Ozonation is an effective way to stabilize new kinds of fermentation media used in the biotechnological production of liquid fuel additives.^10^ Ethanol obtained in this way is relatively inexpensive and can be used as a fuel or fuel additive. Hydrolysates of sucrose are also being considered as alternative raw materials for the production of biodegradable plastics,^11^ fuels or fuel bio-components.^12^

## Sucrose and waste biomass from processing sugar beets as raw materials in biotechnology

**Fig. 2:**
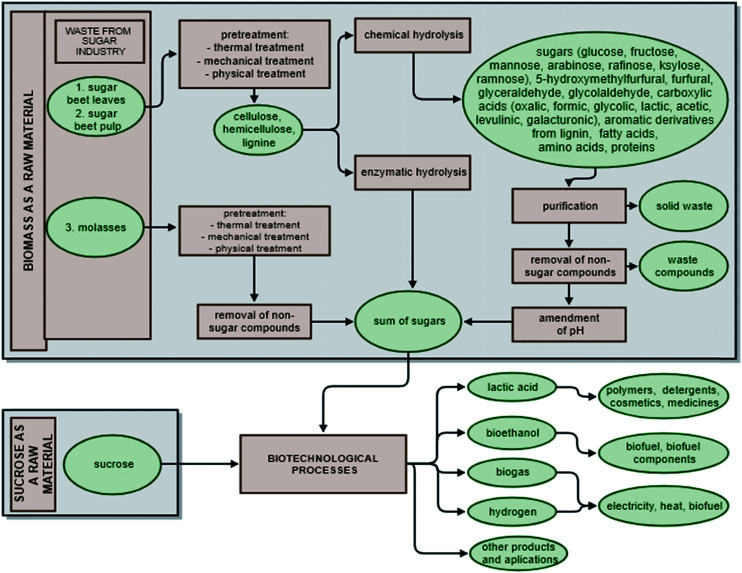
Usage of sugars derived from biomass and crystalline sucrose in biochemical processes.

In biotechnology, both biomass and sucrose are usually processed using one of three methods: anaerobic digestion, fermentation or enzymatic reactions.^13–15^ Depending on the process, it is possible to produce biogas (containing principally CH_4_, CO_2_ and N_2_, H_2_S, NH_3_), biogas rich in hydrogen, bioethanol, biobutanol and lactic acid.^16–19^ The biotechnological transformation of biomass requires appropriate pre-treatment, involving mechanical, physico-chemical, enzymatic or chemical steps, to obtain a mixture of sugar products that can serve as a microbiological medium. The result of enzymatic hydrolysis is a complex mixture of sugars. In the case of chemical hydrolysis, non-sugar products, which are often fermentation inhibitors, are also included in the hydrolyzate (Fig. 2). The use of sucrose in biotechnological processes, on the other hand, does not require costly pre-treatment steps (such as grinding, heating, sonication or ozonation). It is possible to convert sucrose directly without pre-treatment, since sucrose is obtained in the sugar factory as a pure substance suitable for direct conversion in biotechnological processes. Moreover, sucrose, which is the main sugar in fermentable juices, is readily broken down into glucose and fructose by invertase in the periplasmic space of microorganisms during the early stage of fermentation.^20^

Despite its great potential, it is often economically unviable to use sucrose as a raw material in biotechnological processes. In order to reduce costs, intermediates of white sugar production, such as thin or thick sugar beet juices, can be used instead of crystallized sucrose.^21^ However, their use in biotechnological processes requires preliminary purification from non-sugar compounds, such as by ozonation^10^ or adsorption on active carbon.^22^

### Biogas production

Processing 1 ton of sugar beet roots leads to the production of about 70 kg of dry matter – sugar beet pulp.^23^ Annually, 14 million tons of such bio-waste is generated during the production of white sugar in the EU.^24^ Biogas can be produced through the anaerobic digestion of sugar beet pulp. This is a biological process, whereby organic matter is decomposed in the absence of oxygen, generally by placing the appropriate amount of biomass in a specially designed reactor (fermentation chamber) for several days. The contents of the reactor should be maintained at an elevated temperature (37–60 °C), the pH of the medium should be set to an appropriate level, the components should be mixed continuously at an optimal speed. The composition of the biogas which is evacuated should be monitored periodically.^25^ During anaerobic digestion, the effectiveness of biogas production depends on multiple factors, including the construction of the fermentation chambers, technological parameters and the chemical composition of the substrate which feeds the biomass-decomposing microorganisms.^26^ The rate of biogas production is limited primarily by the decomposition of polysaccharides contained in the biomass.^27^ An additional factor that decreases the yields from biogas synthesis is the presence of lignin and crystalline cellulose, which restricts the access of hydrolyzing enzymes to cellulose and hemicelluloses.^28^ Given these limiting factors, the raw material must be subjected to various forms of pretreatment (thermal, pressure and/or mechanical). This task is difficult and requires additional effort, time and energy.^27^ However, in the case of biogas production from beet pulp, the yield is so high that decomposition can be a cost-effective method.^29^

### Hydrogen production

Hydrogen is also of increasing interest as a clean and environmentally friendly energy source. Hydrogen can be produced biologically by many organisms, including green algae,^30^ cyanobacteria,^31^ photosynthetic bacteria^32^ and fermentative bacteria.^33^ The latter can use different sugars, such as starch, cellobiose or sucrose. Moreover, the rate of hydrogen production by fermentation is always higher than that by photosynthesis.^34^ One of the more common processes which use microflora to obtain hydrogen from sucrose is dark fermentation.^35,36^ This anaerobic process for the decomposition of organic matter consists of three main stages: hydrolysis, methanogensis and acidogenesis. During the first two stages, hydrogen (the main product) and fatty acids (by-products) are formed. The resulting organic compounds are used in the next stage for the formation of methane. Hydrogen is created mainly during the first 2 days of the process, while methane production can take up to 20 days. If both hydrogen and methane are collected at the end of each stage, this is referred to as two-step fermentation; if only hydrogen is collected, it is called one-step fermentation.^37^ Fermentation can be carried out under different temperature conditions (from 25 °C to more than 80 °C) or with various substrate concentrations.^38^ However, the critical factor for achieving a high hydrogen production yield is the pH.^39^ Dark fermentation can produce up to 2.8 mol of H_2_ per mol of consumed hexose after 12 hours (the hydrogen content in the resulting biogas is in the range of 48–50%).^40^

Unfortunately, bio-hydrogen is not yet competitive as an energy source against traditional fossil fuels. Most efforts to lower production costs in comparison with fossil fuels have focused on using waste from various industries as raw material. The production of bio-hydrogen now poses few challenges, but technical aspects (such separation of pure hydrogen from gas mixture) and the distribution of the finished fuel (storage, transport) require further development. If suitable solutions could be found, this could lead to alternative fuels becoming economically viable.^41^

### Bioethanol production

Bioethanol is a promising fuel, and can be produced from a variety of renewable sources. Depending on the kind of raw material used, it is differentiated into three generations: first, second and third. First-generation raw materials include corn, wheat, potatoes, fruit and sugarcane, from which the sugars are extracted and subsequently fermented.^42^ Sugars, such as sucrose or starch (which are the main sugars in fermentable juices), are readily broken down in the first step of fermentation into monosaccharides, by invertase in the microorganisms: yeast or bacteria.^21^ Unfortunately, although the process of producing ethanol from these sugars is well established, it is still nearly twice as expensive as the production of gasoline from crude oil. Less expensive bioethanol can be produced directly from the juices of free-sugar containing crops, such as sugarcane, sugar beet or sweet sorghum. However, these substrates are not microbiologically stable, and the fermentation media requires thermal sterilization or ozonation before fermentation.^10^ Bioethanol can also be converted into more expensive petrochemical products, such as butanol and higher homologues, which could provide alternatives to petrol.^43^

The production of first-generation raw materials takes up agricultural space and uses crops that could otherwise be consumed as food. It also requires a large amount of water.^44^ Second-generation biofuels, in which the raw material is non-consumable lignocellulosic biomass, have therefore been developed. Unfortunately, lignocellulosic biomass is not as easy to process as first-generation feedstock, and requires multi-step processing which increases the final cost of the bioethanol produced.^45^ Each step – pre-treatment, hydrolysis, fermentation and distillation – requires an input of energy and residue disposal.^46^ Third-generation bioethanol production uses microorganisms, especially algae, and promises many benefits over the previous generations. Unfortunately, third-generation methods are still very expensive, and commercialization faces many difficulties.^47^ For this reason, the production of bioethanol using algae is being mainly researched at the laboratory scale.^48^

Regardless of the raw material from which sugars (sucrose, glucose, fructose, *etc.*) are derived, they can be converted into bioethanol by yeast (such as *Saccharomyces carlsbergensis*)^49^ or other microorganisms through fermentation, without the need for the sugar to be pre-treated.^48^ As a result of the activity of appropriate enzymes, the saccharides are broken down into simple sugars, which are then fermented to produce ethanol. Anaerobic bacteria capable of converting glucose, fructose and sucrose to ethanol, such as *Zymomonas mobilis*, can be used for this purpose. Many factors can affect the proper functioning of bacteria, including the ethanol that is produced. The resulting ethanol may inhibit the action of the microorganisms, and it is therefore necessary to remove it continuously from the system – by evaporation, selective adsorption or simply by extraction into the organic phase.^50^

The great advantages of using microorganisms in biochemical processes include the high enzyme selectivity and the mild reaction conditions. It is therefore possible to produce the products desired with extremely high yields. Unfortunately, lignin cannot be easily processed biochemically, so lignocellulosic raw materials, used for the production of bioethanol, require pre-treatment such as thermal, acid or enzymatic hydrolysis. Due to its complexity, the biochemical conversion of lignocellulosic biomass into ethanol is still not economically viable, because the price is higher than that of bioethanol obtained through the fermentation of first-generation raw materials. Research should therefore focus on increasing the efficiency of the initial decomposition stage, on reducing the cost of using enzymes and on improving their reusability. It is also important to minimize the costs of pre-treatment, hydrolysis and fermentation, and to make the whole process work continuously.^51^

### Lactic acid production

**Fig. 3:**
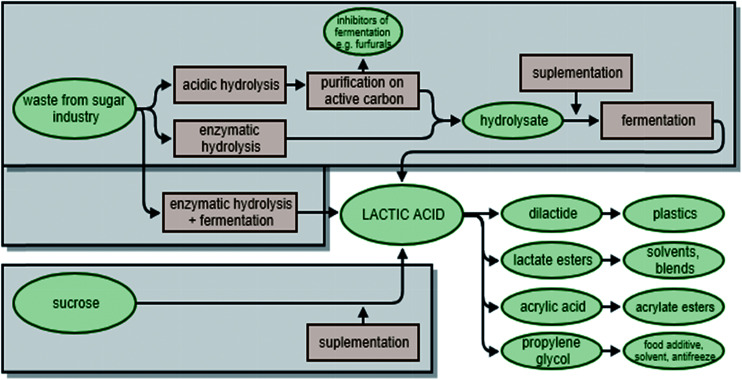
Usage of sugars derived from biomass and crystalline sucrose in lactic acid production.

Another compound that can be obtained biotechnologically from sugars is lactic acid (LA). Lactic acid is used as an acidulant, flavouring and preservative metabolite in many industries, including the food, pharmaceutical, leather and textile industries.^52^ This chemical is also of interest as a chemical platform, and can be subject to multiple transformations. Both small compounds, such as propylene glycol^22,53,54^ or acrylic acid,^55,56^ and high molecular weight polymers, such as biodegradable poly(lactic acid) polymers can be produced from LA (Fig. 3).^57^

Lactic acid is produced at the industrial scale *via* the fermentation of saccharides (*e.g.* glucose) using LA bacteria. However, certain studies have also demonstrated the possibility of using agricultural by-products. Inexpensive raw materials, such as starch or molasses, have been used to replace pure sugars in LA production processes.^58^ Molasses are one of the by-products from the sugar industry that can be used for lactic fermentation. This substratum, rinsed from sugar beet roots after sucrose extraction, consists of 30–50% sugars (mainly sucrose), as well as vitamins, nitrogen compounds and other micronutrients. The high content of sugars in the molasses makes it a good fermentation medium for different kinds of bacteria capable of lactic fermentation (Table 1), such as *Lactobacillus bulgaricus*^59^ or *Lactobacillus casei*.^60^

**Table 1:** Molasses as a raw material for lactic acid (LA) production

Raw material	Microorganism	LA yield [g g^−1^]	Fermentation time [h]	Productivity [g L^−1^ h^−1^]	Article
Cane molasses	*Bacillus coagulans*	0.88	78	2.1	200
Corncob molasses	*Bacillus* sp. *strain*	0.38	48	0.50	200
Cane molasses	*Lactobacillus delbrueckii Uc-3*	0.95	40	4.15	201
Beet molasses	*Lactobacillus delbrueckii IFO 3202*	0.77	—	4.83	202
Sugar molasses	*Enterococcus faecalis RKY1*	0.5	15	4.3	203

Lignocelulose biomass, such as sugar beet pulp, is another widely available by-product of the sugar industry, and is considered as a potential source of sugar for lactic fermentation. The dry basis of sugar beet pulp is composed mainly of polysaccharides, including 22–24 wt% cellulose, 30 wt% hemicelluloses and 15–25 wt% pectin, along with small amounts of fat, protein, ash and lignin at 1.4 wt%, 10.3 wt%, 3.7 wt% and 5.9 wt%, respectively.^61^ Enzymatic or acidic hydrolysis of this by-product leads to the formation of a mixture of monosaccharides, consisting of glucose, fructose, xylose, mannose, galactose and arabionose as well as galacturonic acid (Table 2). Lactic acid bacteria convert the available saccharides directly and selectively into LA (homofermentative conversion) or produce by-products such as carbon dioxide, acetic acid, acetaldehyde and ethanol (heterofermentative transformation). Depending on the need and preferred properties of the final fermentation product, suitable strains of bacteria should be selected for the fermentation of sugars.^62^ The main advantage of producing LA using microorganisms compared to chemical production^63^ is the possibility of obtaining pure acid stereoisomers. In the chemical process, a racemic mixture is always obtained that is optically inactive.^64^

**Table 2:** Composition of sugar beet pulp medium during enzymatic hydrolysis (0.1 mL of Viscozyme and 0.1 mL Ultraflo Max (Novozymes)/50 mL) and acidic hydrolysis (2% H_2_SO_4_)

Sugars	Carbohydrate concentration (g L^−1^) after enzymatic hydrolysis			Carbohydrate concentration (g L^−1^) after acidic hydrolysis	
	4 h	10 h	16 h	130 °C	140 °C
Glucose	18.61 ± 0.70	21.79 ± 0.54	29.74 ± 1.19	2.46 ± 0.08	2.56 ± 0.10
Fructose	4.52 ± 0.40	8.90 ± 0.29	12.46 ± 0.60	2.52 ± 0.30	0.95 ± 0.15
Mannose	3.04 ± 0.14	5.97 ± 0.17	7.04 ± 0.45	1.67 ± 0.12	1.29 ± 0.10
Arabinose	1.54 ± 0.50	2.60 ± 0.87	3.47 ± 0.82	10.81 ± 0.58	13.06 ± 0.62
Galactose	2.27 ± 0.90	3.90 ± 0.39	5.18 ± 0.31	9.39 ± 0.50	13.15 ± 0.65
Rhamnose	0.88 ± 0.59	1.75 ± 0.08	2.26 ± 0.30	4.62 ± 0.42	4.43 ± 0.45
Xylose	0.39 ± 0.053	0.48 ± 0.038	0.47 ± 0.049	1.12 ± 0.08	1.81 ± 0.07
Galacturonic acid	3.66 ± 0.24	5.51 ± 0.44	7.81 ± 0.19	0.48 ± 0.12	2.48 ± 0.32

The cost of raw materials is one of the key factors that determine the economic viability of fermentation processes. Pure glucose, sucrose and starch are expensive feedstocks for the production of LA. Their replacement with inexpensive industrial waste from sugar processing could cut the costs of LA production. Moreover, finding economical and environmentally-friendly uses for by-products of food processing furthers the aims of sustainable development in the food industry.

## Sucrose and waste biomass from processing of sugar beets as raw materials in chemistry

Sugar compounds differ in terms of their chemical structure. Their construction permits synthesis and transformation *via* several reaction groups. Sugars are mostly processed by the food industry, but their range of applications is currently extending considerably, including into energy production.

Monosaccharides, such as ketoses and aldoses, show mutarotation, as a result of which their cyclic forms can change from one into another, by creating hemiacetals.^65^ The mechanism of this pseudo-first order reaction is thought to go *via* the formation of aldehyde. The formation of free aldehyde has been confirmed by polarographic studies.^66^ The mutarotation process has also been the subject of theoretical research using quantum mechanical methods.^67,68^ Theoretical calculations have shown that mutarotation is assisted by solvents and that the first molecule acts as a catalyst, playing a crucial role in the reaction.^69^ Monosaccharides can be obtained from various waste products of the sugar, agricultural and food industries. After purification and separation from interfering compounds, monosaccharides can be subjected to various chemical processes.

**Fig. 4:**
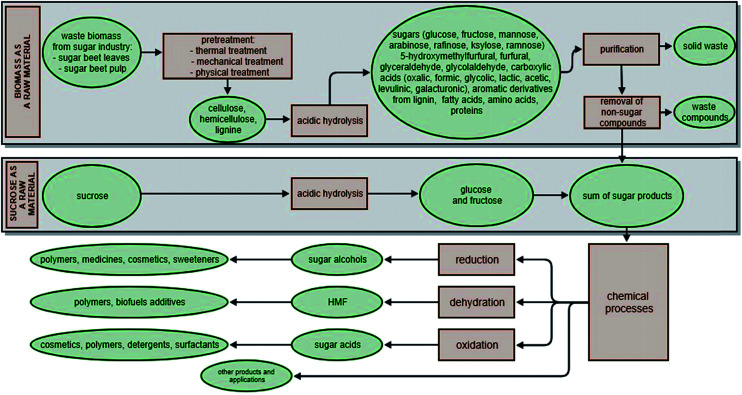
Usage of sugars derived from biomass and crystalline sucrose in chemical processes.

In many cases, the use of crystalline sucrose avoids the need for pre-treatment. Sucrose obtained from sugar factories is a pure compound (99.9%). As a consequence, it is not necessary to remove other substances (such as lignocellulosic compounds) in subsequent processes, or to use pre-purification procedures. Sucrose can be converted easily into monosaccharides by hydrolysis using acid or heterogeneous catalysts (Fig. 4).

### Oxidation of sugars

One of the chemical reactions in which sugars are processed is oxidation. This process has been improved to increase selectivity through the use of suitable catalysts. As a result of catalytic oxidation of sugars, aldonic, aldaric and uronic acids^70^ are produced (among others), and these are widely used in the food, cosmetics, pharmaceutical and medical industries.^71^ Aldonic acids are obtained by oxidation of the aldehyde group, whereas dicarboxylic aldaric acids are produced by the simultaneous oxidation of the aldehyde group and the terminal hydroxyl group. Uronic acids require selective oxidation of the terminal hydroxyl group only, which can be achieved using appropriate enzymes.

Homogeneous catalysts, such as nitric acid, can be used for the oxidation of sugars.^72^ In the case of acid catalysis, different acids can be used. With each acid, the reaction proceeds slightly differently, but always involves a direct oxidant attack on the available carbonyl group.^73^ In a base catalyzed reaction, the enediol form is created as an intermediate product during oxidation of reducing sugars. The main advantages of these processes are the ease with which the catalyst can be separated from the reaction mixture and the higher selectivity of the transformations, resulting in fewer by-products. The use of stable and highly selective heterogeneous catalysts enables the chemical synthesis of aldonic acids under mild conditions. Such processes are environmentally friendly and competitive with traditional chemical or more expensive enzymatic methods.

Mechanisms for the catalytic oxidation of sugars have been the subject of research for many years.^74–76^ Noble metals (Pt, Pd, Au, Ru)^77–80^ or bimetallic systems (Ag/Au, Au/Pt, Ru/Bi, Pd/Te, Pd/Bi, Pd/Tl^81–85^*etc.*) are used as catalysts. In the case of bimetallic systems, the addition of a second metal increases selectivity and the activity of the entire catalytic system.^83,86^ The catalytic properties of bimetallic systems depend on the structure and composition of the surface, which in many cases is different from that of the bulk material. Two metals may create intermetallic compounds on the surface (*e.g.* Pd/Bi, Pd/Tl, Pd/Te^87^ systems) or intermetallic alloys (*e.g.* Pd/Ag, Pd/Au).^88^ It is well known that when two metals form an alloy, the surface will be enriched with the metal that has the lower surface of sublimation. Many works^89^ clearly show that intermetallic interactions in Pd/M catalysts play an important role in oxidative dehydrogenation of aldoses into aldonic acids. Knowing exactly which type of intermetallic structure forms on the surface may be key to understanding the function of metal promotors in this process.

Nitric acid can also be used for the preparation of aldaric acids. This reaction has been known since the 1880s.^90^ However, due to its low conversion efficiency, unfavourable reaction enthalpy and harmful by-products (nitric oxides), the process has not been fully commercialized and has required significant industrial improvements.^91,92^ For the preparation of glucaric acid from glucose, metallic catalysts (usually palladium or platinum) are used.^93^ More sophisticated systems are now being developed, containing for example (2,2,6,6-tetramethylpiperidin-1-yl) oxygen (tempo), or 4-acetylamino-tempo, in the presence of which the oxidation reaction can proceed chemoselectively.^94^ Aldaric acids can also be synthesized from the corresponding uronic acids using gold catalyst or electrolytic oxidation.

In chemical synthesis, uronic acids are derived from *O*-glycosides and *O*-furanosides. Aldoses may also be used, but in this case it is necessary to protect the secondary hydroxyl groups in order to selectively oxidize the primary group.^95^ Such oxidation is performed by KMnO_4_ or in the presence of a metallic catalyst, such as platinum.^96,97^

Sugar acids have a wide variety of possible applications. Aldonic acids are used in the food or agriculture industries (for the removal heavy metals from water or soil); in cosmetics (as anti-microbial agents) and in the plastics industry (as silicone surfactants). Uses of uronic acids include in biomedicine, as precursors of polymers; aldaric acids are used as corrosion inhibitors; cross linkers are used in hydrogels; and monomers are used in the production of plastics.^98^

### Dehydration of sugars

**Fig. 5:**
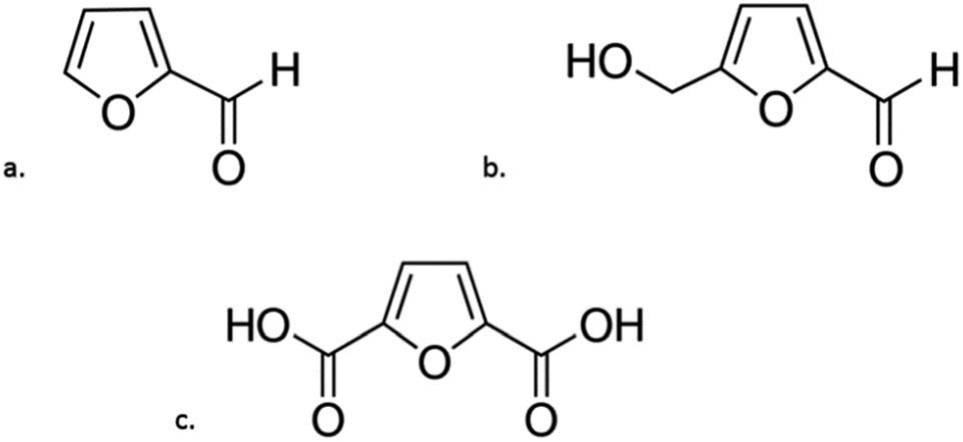
Chemical forumulas of: (a) furfural; (b) 5-hydroxymethylfurfural; (c) 2,5-furandicarboxylic acid.

Dehydration is another group of chemical reactions by which sugars can be converted. The process of sugar dehydration has been developed since the beginning of the 19^th^ century. At present, the process is widely used for the synthesis of furan compounds, which can be transformed into many valuable chemicals, including fuels and fuel components.^99^ The most promising compounds in this group are furfural (F), 5-hydroxymethylfurfural (HMF) and 2,5-furandicarboxylic acid (FDCA) (Fig. 5).

Today, one of the most important products of this reaction is HMF^100,101^ which has been the subject of hundreds of studies, as it is a chemical platform for obtaining many valuable compounds. HMF is formed by the thermal decomposition of sugars, and is therefore a naturally occurring compound in honey, coffee, juices, wine, bread and other foods. One of the processes that causes its formation is caramelization. This reaction takes place under different conditions, depending on the sugar. For instance, for fructose it begins at 110 °C; for other hexoses it may be necessary to heat the mixture to 160 °C or higher.^102^ The second type of reaction that results in HMF is the Millard reaction.^103,104^ The mechanism of this reaction is two-step. In the first step, the reducing sugar reacts with the amino acid, resulting in the so-called Amadori compound. The second step is the removal of the amino acid and the formation of 3-deoxyglucosone (3-DG). This compound is highly reactive, and its degradation leads to the formation of HMF, among other compounds. The mechanism of HMF formation depends strongly on the type of solvent used. Solvents such as dimethyl sulfoxide (DMSO) and ionic liquids can play an active role in the reaction of sugar conversion to HMF, altering the mechanism. Difficulties arise mainly from the inability to investigate the intermediates and the wide range of by-products generated. By-products of this reaction include organic acids, furan derivatives and polymeric compounds (including humins). Typically, when the HMF synthesis reaction is prolonged the yield of HMF decreases and levulinic^105,106^ and formic acids form as by-products.^107^

During typical acid dehydration of sugar, HMF is formed mainly from fructose, as described in the literature.^108–111^ Obtaining this compound from glucose requires isomerization of glucose to fructose, because the yield of HMF formation from glucose is much lower.^112,113^ The isomerization of glucose, associated with the subsequent dehydration of fructose, is quite a challenging step, because the first process is catalyzed by the base while the second is catalyzed by an acid. In order to achieve this result, single phase,^106,114–116^ biphasic^117,118^ or ionic liquid systems are used.^119,120^ The single phase system is the oldest and the most popular. The different reactivity of glucose in comparison to fructose, and the higher selectivity of fructose to HMF, is explained by the greater stability of the fructose ring. The more stable fructose ring allows the formation of an intermediate enediol form which subsequently converts to HMF.^121^

**Fig. 6:**
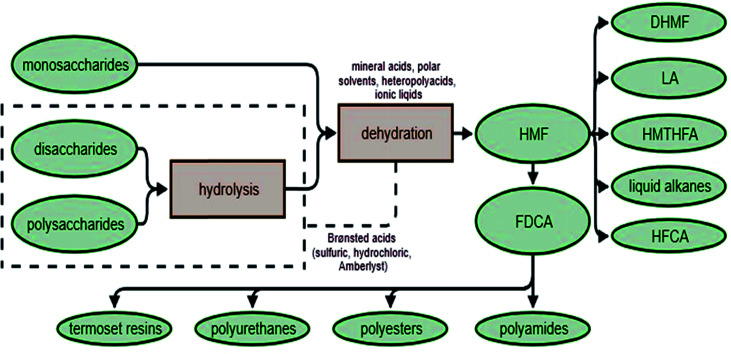
Synthesis and applications of HMF (HMF: 5-hydroxymethylfurfural; FDCA: 2,5-furandicarboxylic acid; DHMF: 2,5-dihydroxymethylfuran; LA: levulinic acid; HMTHFA: 5-hydroxymethyltetrahydrofurfural; liquid alkanes; HFCA: 5-hydroxymethylfuranoic acid).

HMF can be obtained not only through the dehydration of monosaccharides,^122–124^ but also from di- and polysaccharides.^125–127^ The first step is the hydrolysis of polysaccharides. This reaction is faster than dehydration,^128,129^ which allows compounds such as sucrose or inulin to be converted into HMF in a single batch reaction.^130^ If glucose is the main sugar in the reaction mixture, isomerization to fructose is necessary before dehydration. The yield and selectivity to HMF in direct sugar conversion are rather low, and increasing the productivity of this reaction is the subject of much scientific research. Obtaining HMF, a valuable chemical compound, from sugars (or even better, from biomass) could become be a very cost-effective process, allowing the conversion of waste materials into valuable feedstock, which might then be used for the production of polymers, biofuels or energy (Fig. 6).

In the production of HMF from polysaccharides and biomass, a variety of different homo- or heterogenic catalytic systems are used. Following hydrolysis and dehydration processes, HMF can be catalyzed simultaneously by Brønsted acids (*e.g.* HCl, H_2_SO_4_, and Amberlyst), while the use of Lewis acids (*e.g.* AlCl_3_, CrCl_3_, and Sn-beta) leads to sugar isomerization. The reactions can be carried out in a variety of solvents, including water and polar aprotic solvents (*e.g.* dimethyl sulfoxide, tetrahydrofuran and methyl isobutyl ketone), which increase the HMF yield by continuously eliminating the product from the reaction mixture or by limiting the formation of byproducts.^131,132^ Recently, systems using ionic liquids or heteropolyacids have attracted interest. For example, it is possible to obtain HMF in ionic liquids with catalysts such as CrCl_2_/HCl in 1-methyl-3-octylimidazolium chloride, ZnCl_2_/HCl,^133^ CrCl_2_, SnCl_4_ in 1-ethyl-3-methylimidazolium tetrafluoroborate ([EMIM]BF_4_)^134^ or [AEMIM]BF_4_ in DMSO.^135^ Other catalytic systems based on heteropolyacids used in the production of HMF include (HOCH_2_CH_2_N(CH_3_)_3_)_*x*_H_3_–*x*PW_12_O_4_0 (Ch_*x*_H_3_–*x*PW_12_O_40_, *x* = 1, 2 and 3) in a double solvent system containing methyl isobutyl ketone (MIBK) and H_2_O,^136^ a Brønsted–Lewis-surfactant-combined heteropolyacid (HPA) Cr[(DS)H_2_PW_12_O_40_]_3_ (ref. 137) or cesium salt of dodecatungstophosphoric acid (Cs_2.3_H_0.7_PW_12_O_40_) in a dimethyl sulfoxide (DMSO)/water mixed solvent.^138^

**Fig. 7:**
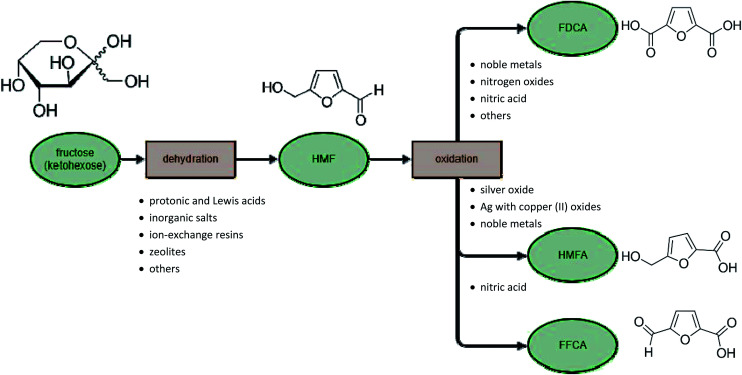
Catalytic oxidation of HMF into acids.

HMF can be converted in several ways, depending on the reaction type of the functional groups. The formyl group may be oxidized easily to the carboxylic group: 5-hydroxymethyl-2-furancarboxylic acid (also named 5-hydroxymethyl-2-furoic acid, HMFA) is selectively obtained from HMF without reaction of the hydroxyl group.^139–141^ A second possibility is oxidation of both the hydroxylic and formyl groups to produce 2,5-furandicarboxylic acid (this process will be described in a later section). Oxidation of HMF is also possible with only the hydroxyl group, which transforms HMF into 5-formyl-2-furancarboxylic acid (FFCA). The latter two processes occur through different numbers of reactions, alongside each other, depending on the conditions. In acidic media (aquatic or in a mixture of solvents such as DMSO and acetic acid) it is possible to maintain the carboxylic group and stabilize the formyl group (Fig. 7).^142^

Because HMF has enormous productive potential in the chemical industry, researchers are constantly looking for more efficient systems for obtaining this compound from sugars. Moreover, its market price is many times higher than the materials from which it can be produced. Currently, the dominant factor that limits the profitability of HMF is the price of pure fructose, so new ways of obtaining HMF from other saccharides or waste material from industry are being developed. If waste biomass is used as a feedstock, it is possible to obtain substrate for reactions without a large financial outlay. However, the potential for commercial scaling-up, as well as the production yield or substrate conversion efficiency, are considerations when evaluating new systems for HMF production.

### Hydrogenation of sugars

Another important group of chemicals that can be obtained from sucrose or biomass waste are the sugar alcohols. Among these alcohols are distinguished sorbitol, mannitol, xylitol, erythritol, isomalt and hydrogenated starch hydrolysates. These compounds are naturally occurring and they are produced in industry by the hydrogenation of sugars. Sugar alcohols are commonly used in food products, usually as sucrose substitutes, since they have lower calorific value due to their poorer assimilation. Xylitol is the sugar that most resembles sucrose, in terms of sweetness and appearance.^143^

Xylitol is mainly produced by the hydrogenation of xylose in the presence of RANEY® nickel catalyst. The synthesis consists of four stages. In the first step, xylose and other sugars are extracted from hardwood by acid hydrolysis of hemicellulose chains. Then, the mixture of monosaccharides and unreacted cellulose is purified and decolored. In the next step, the hydrolyzate is hydrogenated at a temperature in the range of 80–140 °C, under up to 50 atm of H_2_ pressure in the presence of RANEY® nickel catalyst. Finally, xylitol is separated from the solution by crystallization. The following purification and extraction process is the most costly stage of xylitol production, due to the low content of pure alcohol in the reaction mixture after hydrogenation.^143,144^

Sorbitol is the second most important industrial sugar after xylitol. It can be obtained from fructose and glucose mixtures or directly from sucrose. This alcohol is mainly used as a food additive, successfully replacing traditional sugar due to its greater sweetness and lower caloric value in comparison to sucrose. Sorbitol is also used in other branches of industry: in medicine (for the production of vitamin C, bacterial culture media and medicines^145,146^); in pharmaceutics (as a sweetener in syrups, toothpaste and mouthwashes^147–149^); in the chemical industry (as a chemical platform for the synthesis of many valuable chemicals^150,151^ or in polymerization processes^152–154^) and in cosmetics (in emulsions, moisturizers and lotions^155,156^). Sorbitol can be produced by the hydrogenation of an equivalent mixture of fructose and glucose in a temperature range of 120–160 °C over RANEY® nickel catalyst.^157^ Under these conditions, glucose and beta-fructose are converted into sorbitol while alpha-fructose is reduced to mannitol. As a result, a mixture containing sorbitol and mannitol is obtained in a weight ratio of 75 : 25. It is also possible to use sucrose directly as a substratum, but an additional stage of hydrolysis of sugar into monosaccharides and further reduction is required.

The most expensive step in the production of sorbitol from a mixture of monosaccharides is the separation of the products. To reduce these costs, other materials which can by hydrolyzed to form glucose, such as maize starch or cellulose, are used.^143^ However, these materials should first be pre-treated to weaken the hydrogen bonds that stabilize the substratum. The cellulose can then be hydrolyzed to glucose and hydrogenated. Both reactions, hydrolysis and hydrogenation, are performed using catalysts. Supported precious metals (Ru, Pt and Pd) can be used simultaneously or in combination with mineral acids such as H_2_SO_4_, HCl or heteropolyacids.^158–160^ The process of obtaining glucose through hydrolysis of cellulose requires conditions of high temperature and acidity. It is difficult to obtain a high glucose yield, because under these conditions the degradation of glucose occurs faster than cellulose hydrolysis.^161^ The hydrogenation process is less problematic, since the sugar alcohols (mannitol, sorbitol) obtained are more thermally stable than glucose.

Much research is currently focusing on the development of more efficient methods of cellulose hydrolysis, in which there would be much less loss of glucose. Possibilities include the use of enzymatic hydrolysis, appropriate mineral acids or supercritical water.^150,162–164^ At the moment, the most promising and environmentally-friendly process appears to be the use of supercritical water. This method limits the formation of by-products and takes considerably less time compared to enzymatic or acidic hydrolysis. Supercritical water hydrolysis may also be used for processing waste materials from the sugar industry, such as sugar beet pulp or sugar beet leaves. It has been reported that hydrolysis of sugar beet pulp in supercritical water leads to glucose as the main product of the reaction. Hydrogenation of the resulting monosaccharide over a supported Ru catalyst produces sorbitol.^163^ The production of sugar acids should therefore be considered as one of the possible ways of utilizing sugar waste, as well as surplus sugar.

## Polymers obtained from sugars

Polymers produced from the processing of crude oil are commonly used to manufacture plastics. The resulting compounds are characterized by high strength, resistance to external factors (including chemical resistance), the ability to be moulded easily and the possibility of biological sterilization. These features also make synthetic polymers an almost ideal feedstock for the production of various materials. The main disadvantage of synthetic polymers is the negative impact on the environment. Plastic packaging takes many years to decompose and requires disposal processes that generate additional costs. To counteract or completely eliminate these problems, scientists in collaboration with industry have been seeking alternative chemicals derived from biomass or natural resources, which could allow the synthesis of biodegradable and environmentally-friendly polymers (Table 3). Sucrose would become a raw material for the production of packaging and containers. This would also provide an alternative use for surplus sucrose.

**Table 3:** Potential polymers obtained from sugar and its derivatives

Substrates	Product	Potential applications	Ref.
Dimethyl ester of 2,3:4,5-di-*O*-methylene–galactaric acid + 1,*n*-alkanediols HO(CH_2_)_*n*_OH + catalyst	Poly galactarates (PE–*n*Galx)	Food packaging; medical devices	166
Dimethyl ester of adipic acid + 1,*n*-alkanediols HO(CH_2_)_*n*_OH + catalyst	Polyadipates (PE–*n*Ad)		
Activated galactaric (peracetylated galactaric acid chloride, galactaric acid methyl ester)	Carbohydrate-segmented silicone polyamides	Clinical applications; cosmetics and textile industries	193
Activated glucaric acid derivatives (heterogenous esterification product of glucaric acid)			
2,5-Furandicarboxylic acid (FDCA) + ethylene glycol (EG)	poly(ethylene 2,5-furandicarboxylate) (PEF)	Food packaging, in particular: packaging of soft drinks, water and alcoholic beverages; films; fibers	194
Dimethyl 2,5-furandicarboxylate (DMFDC) + ethylene glycol (EG)			
2,5-Bis(hydroxyethyl)furandicarboxylate + antimony(iii) oxide			195
(DCFDC) + ethylene glycol (EG)			196
FDCA + 1,4-butanediol + titanium(iv) butoxide (Ti(OBu)_*n*_)	Poly(1,4-butylene 2,5-furandicarboxylate) (PBF)	Biodegradable copolymers; packaging	197
Furfural + base + air/O_2_ + catalyst and furan + H_2_			198
Cyclic oligo(alkylene 2,5-furandicarboxylate)s + Sn(Oct)_2_			184
Sorbitol + citric acid + sebacic acid	poly(sorbitol citric sebacate) [p(SCS)]	Potential biomedical applications; materials metabolized in the human body	154
sorbitol + tartaric acid + sebacic acid	poly(sorbitol tartaric sebacate) [p(STS)]		
Aromatic isocyanate (4,4′-di-phenylmethane diisocyanate) + polyols (polytetramethylene glycol, polycaprolactone, polycarbonate diols) + sucrose	Polyurethane elastomer (PUE)	Flexible slab and molded; foam; sold elastomers; carpet backing; heat insulation; tremor insulation; cases for commercial instruments	199

### Sugar acids as a monomer for polymerization

Sugar acids can be used to obtain linear polymers such as polyamides, polyesters, polycarbonates, polyurethanes and polyanhydrides. These polymers are most often obtained *via* condensation polymerization. Prior to the formation of linear polymers, the secondary hydroxyl groups should be protected in order to avoid unwanted by-products.^165^ To synthesize polyesters that are analogous to the common industrial polymers poly(ethylene terephthalate) (PET) and poly(butylene terephthalate) (PBT), the functional groups can be blocked using the dimethyl ester of 2,3:4,5-di-*O*-methylene–galactaric acid (Galx).^166^ Sometimes, aldaric or gluconic acid moieties (such as trimethylsilyl derivatives) are used as polymeric additives (*e.g.* polydimethylsiloxanes) to improve hydrophilicity and biodegradability. In this way, silicone surfactants are created, which have a wide range of applications in the cosmetics and textile industries.^167,168^

Sugar acids can not only be used directly as building blocks for polymers, but also be converted into other monomers. One of the popular monomers that can be obtained from glucaric acid is adipic acid. Adipine acid is usually produced from petrochemical sources.^169,170^ However, this has a negative impact on the environment, so other pathways are now preferred for obtaining this valuable chemical. Reactants which may lead to adipic acid include glucaric acid and muconic acid.^171^ It is also possible to use biomass and biological processes.^172^

Another polymer precursor that can be obtained from sugar or sugar acids, and which is among the most important building-block chemicals, is 2,5-furandicarboxylic acid (FDCA).^173^ The U.S. Department of Energy (DOE) lists twelve such chemical platforms which can be obtained from sugars.^174^ These chemical platforms can be produced *via* biological or chemical conversion and then transformed into a wide range of other high-value substances. The common feature of building-block chemicals is the presence of many functional groups, which have the potential to react with a wide number of other compounds. 2,5-Furandicarboxylic acid can be used in synthesis and reaction pathways, leading to many materials and products used currently by various industries.^175^

### FDCA as a monomer for polymerization

2,5-Furandicarboxylic acid (FDCA) has attracted the attention of scientists as a potential monomer for use in polymerization processes leading to the production of biodegradable biopolymers.^173,176,177^ The increasing interest in this compound is shown by the number of publications on the subject.

**Fig. 8:**
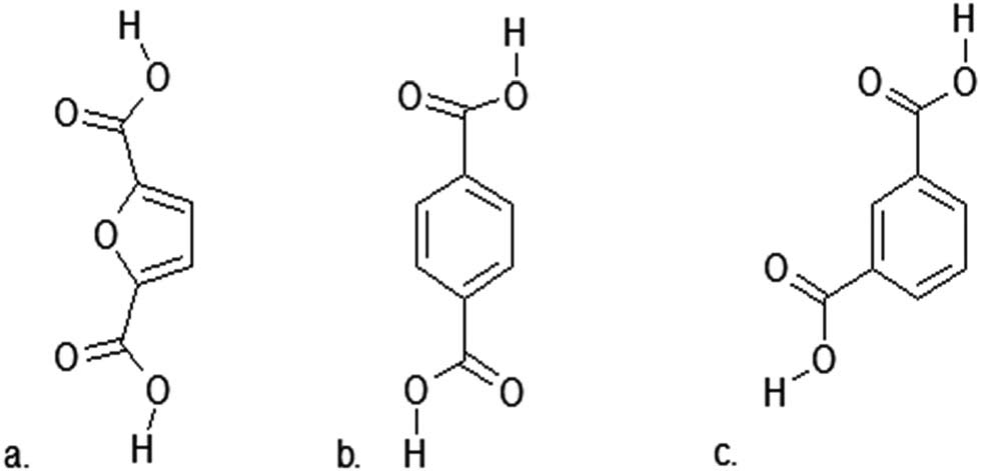
(a) 2,5-Furandicarboxylic acid (FDCA); (b) Terephthalic acid (TPA); (c) isophthalic acid (IPA).

2,5-Furandicarboxylic acid is a promising monomer for use in many polymerization processes, due to its similarity to the petrochemicals terephthalic acid (TPA) and isophthalic acid (IPA) (Fig. 8). 2,5-Furandicarboxylic acid also has the potential to be used to create completely new polymer materials with unique properties.^173^

FDCA can be obtained from sugars (fructose, glucose, sucrose or polysaccharides) *via* oxidation of an intermediate product such as 5-hydroxymethylfurfural (HMF). The compound HMF can be created in one of the three possible ways. The first is by the acid catalyzed dehydration of hexose. The second is through a Maillard reaction in the presence of amino acids and amines. The third is *via* the aldol condensation of three smaller C-3 molecules.^100^ In syntheses designed for the industrial production of HMF (as a precursor of FDCA) the first path is most often used. The next step is the oxidation of HMF to FDCA. This requires suitable catalysts, such as supported noble metals^178,179^ or bimetallic systems^180^ deposited on carbon or metal oxides (such as aluminum, titanium or zirconium and others^181–183^). The general concept of sugar-to-FDCA reactions seems fairly straightforward, but it should be noted that, besides the reactions which lead to the main product, there are a number of side reactions which greatly reduce the yield of the main process.

**Fig. 9:**
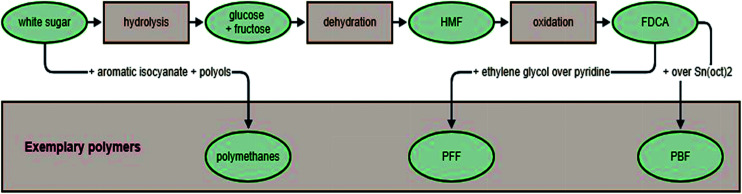
Polymers obtained from sucrose.

If FDCA is obtained with a good yield, then it can be used as a monomer for further polymerization (Fig. 9). Common heteropolymers synthesized from FDCA and other monomers, such as aliphatic and aromatic monomers, include polyesters and polyamides.^173^

**Fig. 10:**
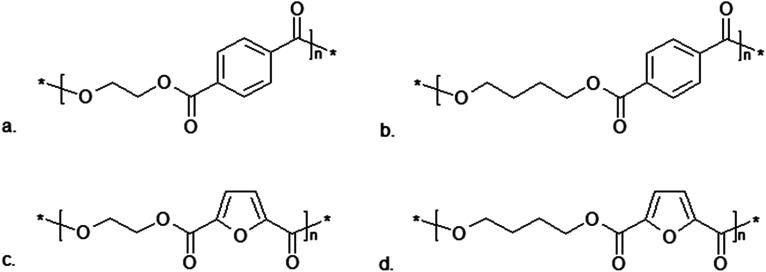
Comparison of the polymer structures obtained from terephthalic and 2,5-furandicarboxylic acid:^184^ (a) poly(ethylene terephthalate) PET; (b) poly(butylene terephthalate) PBT; (c) poly(ethylene 2,5-furandicarboxylate) PEF; (d) poly(butylene 2,5-furandicarboxylate) PBF.

One of the polymers that can be obtained using FDCA is poly(ethylene 2,5-furandicarboxylate) (PEF), which resembles industrial poly(ethylene terephthalate) (PET) in its structure (Fig. 10). 2,5-Furandicarboxylic acid dichloride reacts with ethylene glycol in the presence of pyridine under mild temperature conditions. However, to obtain a higher molecular weight polymer the process requires slight modification. With 1% Sb_2_O_3_ under a high vacuum and with a rapid temperature rise from 70 to 220 °C, bis(hydroxyethyl)-2,5-furandicarboxylate undergoes tranesterification. During this reaction, the viscosity of the solution increases until it becomes a solid. The process is completed once the solution has returned to room temperature.^185^

Another polyester obtained from FDCA is poly(butylene 2,5-furandicarboxylate) (PBF), which is an analogue of poly(butylene terephthalate) (PBT) (Fig. 10). PBF is produced *via* Ring-Opening Polymerization (ROP) catalyzed by tin(ii)2-ethylhexanoate (Sn(Oct)_2_).^184^ This reaction is generally used in the polymerization of cyclic monomers (for example lactones, lactides), when the initiator of the reaction is an alcohol or a hydroxyl group. Due to the initial reaction phase, this method is well-suited for modifying cellulose polymers or their derivatives. The mechanism of this reaction varies depending on the monomer or catalyst used. A commonly-used catalyst is Sn(Oct)_2_, which enables the polymerization of compounds such as ε-caprolactone (ε-CL) or lactide.^186^ This catalyst can also be used in the ROP of cyclic oligo(alkylene 2,5-furandicarboxylate)s at temperatures of 200–220 °C, resulting in PBF (Table 3).

### Sorbitol and sucrose as monomers for polymerization

Biodegradable polymers can also be synthesized using sugar alcohols, such as sorbitol. This chemical meets all the requirements of a monomer for polymerization. Sorbitol is inexpensive and readily available from renewable sources. It also has many functional groups, which allow multiple connections in three-dimensional networks. Its greatest advantage is that it is harmless to humans and completely metabolized into CO_2_.^187,188^

Polycondensation dehydration of alcohols such as sorbitol or glycerol can be performed using carboxylic acids (tartaric or maleic acid). In this regioselective reaction, scandium trifluoromethane-sulfonate is used as a catalyst and polyesters without crosslinks are formed.^189^ It is also possible to synthesize biodegradable polymers without using a catalyst by melt condensation of sorbitol with citric acid, tartaric acid and sebacic acid. This reaction results in poly(sorbitol citric sebacate) and poly(sorbitol tartaric sebacate). These polymers are characterized by random cross linked networks and physical, mechanical and degradation properties that make them suitable for biomedical applications (Table 3).^154^

Sorbitol can also be used for the synthesis of polymers, such as polyurethanes, in the form of oxoethylated or oxylpropylated derivatives. These can be used as additives to improve the thermal stability of the final products and reduce their tendency to oxidize. Polyurethanes are used in the synthesis of synthetic foams, insulation materials, elastomers and sealants. Polyurethane elastomers containing natural substances can be used in the medical industry.^190–192^

The wide availability and high purity of sucrose added to its low production cost makes this natural compound ideal as a polyurethane component, complementing the main polymer chain. Sucrose acts as a crosslinker in one-shot synthesis of polyurethane. Reactions using an aromatic isocyanate (4,4′-di-phenylmethane di-isocyanate), polyols including a polyether polyol(polyteramethylene glycol) and two polyester polyols(polycaprolactone and polycarbonate diols) lead to polyurethane elastomers. The polyurethanes obtained by this method are transparent and, with the addition of sucrose, the hydrogen bonds with the urethane units, increasing their hardness. The presence of sucrose in the polyurethane chain improves the stability and chemical resistance of materials, thus their potential for use in medicine is increased (Table 3).^199^

## Conclusions

Given its relatively high price and because there was no surplus production, white sugar was not until recently considered as a raw material for synthesis. However, changing market conditions are forcing European sugar producers to find alternative uses for sucrose, to sustain production levels and profitability. With the lifting of limits on sugar beet production in the European market and white sugar prices likely to fall, there are opportunities for the development of new chemical technologies based on the products of sugar beet processing (sucrose, melase and waste materials).

White sugar can be used in many biotechnological processes, but also can be considered an interesting substratum for chemical synthesis. Currently, there are only a few processes in the chemical industry that use sucrose as a raw material. The production of biodegradable polymers from dehydrated monosaccharides obtained from sucrose or polysaccharides found in waste biomass seems an especially promising solution. The most efficient ways of producing functionalized materials from sugar are through the acidic hydrolysis of sucrose or waste biomass, *via* the catalytic dehydration of monosaccharides to HMF, followed by catalytic oxidation to FDCA and polymerization to biodegradable polymers with the required functional properties. The introduction of technologies in which sucrose is a chemical raw material is of interest both to industry and science, as manifested by the growing number of research publications on this subject.

The sugar industry is particularly interested in obtaining biodegradable polymers from sugar beet biomass. In Poland, a project is being implemented, co-financed by the National Centre for Research and Development, which will allow the building of a quarter-scale installation for obtaining and dehydrating to HMF monosaccharides from the catalysis of waste sugar beet biomass. Alternatively, in our prototype installation, the process of acidic hydrolysis of waste biomass to furfural and monosaccharides, which may subsequently be processed to obtain products (such as fuel additives from furfural)^204,205^ or used in biotechnological processes (for example, to obtain protein feed from yeast, bioethanol or lactic acid).^206,207^ It should be emphasized that most of the concepts for chemical processes which have been presented here for utilizing products of sugar processing use homo- and heterogeneous catalysts. It seems that in modern technological concepts, biotechnology and catalytic processes should be combined to increase the profitability and efficiency of commercial solutions.

## Conflicts of interest

There are no conflicts to declare.

## Supplementary Material

## References

[cit1] Maitah M., Řezbová H., Smutka L., Tomšík K. (2016). Sugar Tech.

[cit2] Szajner P. , Wieliczko B., Wigier M., Hamulczuk M. and Wrzaszcz W., Research for Agri Committee – the Post-Quotas Eu Sugar Sector, 2016

[cit3] Brouns F. (2015). Agro Food Ind. Hi-Tech.

[cit4] Guven C. and Polet Y., EU-28 Sugar Annual Report, 2015

[cit5] Aguirre E. K., Mytton O. T., Monsivais P. (2015). BMJ [Br. Med. J.].

[cit6] Cooke D. A. and Scott R. K., The Sugar beet crop: science into practice, Chapman & Hall, 1993

[cit7] Parawira W., Murto M., Read J. S., Mattiasson B. (2005). Process Biochem..

[cit8] FAO Investment Centre Division , Agribus. Handbooks, 2009, pp. 1–55

[cit9] Finkenstadt V. L. (2014). Sugar Tech.

[cit10] Dziugan P., Balcerek M., Binczarski M. J., Kregiel D., Kucner M., Kunicka-Styczynska A., Pielech-Przybylska K., Smigielski K., Witonska I. A. (2016). Biotechnol. Biofuels.

[cit11] Vijayendra S. V. N., Shamala T. R. (2014). Crit. Rev. Biotechnol..

[cit12] Sheldon R. A. (2016). J. Mol. Catal. A Chem..

[cit13] Montañés R., Pérez M., Solera R. (2014). Chem. Eng. J..

[cit14] Narra M., Divecha J., Shah D., Balasubramanian V., Vyas B., Harijan M., Macwan K. (2017). J. Environ. Chem. Eng..

[cit15] Hernández D., Riaño B., Coca M., García-González M. C. (2015). Chem. Eng. J..

[cit16] Ojeda K., Kafarov V. (2009). Chem. Eng. J..

[cit17] Moshi A. P., Temu S. G., Nges I. A., Malmo G., Hosea K. M. M., Elisante E., Mattiasson B. (2015). Chem. Eng. J..

[cit18] He C.-R., Huang C.-L., Lai Y.-C., Li S.-Y. (2017). J. Taiwan Inst. Chem. Eng..

[cit19] Oonkhanond B., Jonglertjunya W., Srimarut N., Bunpachart P., Tantinukul S., Nasongkla N., Sakdaronnarong C. (2017). J. Environ. Chem. Eng..

[cit20] Dodić S., Popov S., Dodić J., Ranković J., Zavargo Z., Jevtić Mučibabić R. (2009). Biomass Bioenergy.

[cit21] Dziugan P., Balcerek M., Pielech-Przybylska K., Patelski P. (2013). Biotechnol. Biofuels.

[cit22] Berlowska J., Binczarski M., Dudkiewicz M., Kalinowska H., Witonska I. A., Stanishevsky A. V. (2015). RSC Adv..

[cit23] Spagnuolo M., Crecchio C., Pizzigallo M. D. R., Ruggiero P. (1997). Bioresour. Technol..

[cit24] Altundogan H. S., Bahar N., Mujde B., Tumen F. (2007). J. Hazard. Mater..

[cit25] Suhartini S. , The anareobic digestion of sugar beet pulp, PhD thesis, University of Southampton, 2014

[cit26] Markowski M., Białobrzewski I., Zieliński M., Dębowski M., Krzemieniewski M. (2014). Renewable Energy.

[cit27] Ziemiński K., Kowalska-Wentel M. (2017). Appl. Biochem. Biotechnol..

[cit28] Frigon J.-C., Guiot S. R. (2010). Biofuels, Bioprod. Biorefin..

[cit29] Swiniarski K. , Sugar beet pulp as a substrate for the production of biogas Profitability of investments in biogas plants, AV Akademikerverlag, 2013

[cit30] Chia S. R., Chew K. W., Show P. L., Ong H. C., Phang S.-M., Ling T. C., Nagarajan D., Lee D.-J., Chang J.-S. (2017). Renewable Energy.

[cit31] Singh V., Chaudhary D. K., Mani I., Dhar P. K. (2016). Renewable Sustainable Energy Rev..

[cit32] Lu C., Zhang Z., Ge X., Wang Y., Zhou X., You X., Liu H., Zhang Q. (2016). Int. J. Hydrogen Energy.

[cit33] Xia A., Jacob A., Herrmann C., Murphy J. D. (2016). Energy.

[cit34] Das D. (2001). Int. J. Hydrogen Energy.

[cit35] Tao Y., Chen Y., Wu Y., He Y., Zhou Z. (2007). Int. J. Hydrogen Energy.

[cit36] Lin C. Y., Lay C. H. (2005). Int. J. Hydrogen Energy.

[cit37] Liu D. , Kgs. Lyngby: DTU Environment, 2008

[cit38] Cardoso V., Romão B. B., Silva F. T. M., Santos J. G., Batista F. R. X., Ferreira J. S. (2014). Chem. Eng. Trans..

[cit39] Craven S. E. (1988). J. Food Prot..

[cit40] Lutpi N. A., Md Jahim J., Mumtaz T., Harun S., Abdul P. M. (2016). Process Biochem..

[cit41] Khan M. A., Ngo H. H., Guo W., Liu Y., Zhang X., Guo J., Chang S. W., Nguyen D. D., Wang J. (2017). Renewable Energy.

[cit42] Jacques K. A. , Lyons T. P. and Kelsall D. R., The Alcohol Textbook. A reference for the beverage, fuel and industrial alcohol industries, Nottingham University Press, 3rd edn, 1999

[cit43] Dziugan P., Jastrzabek K. G., Binczarski M., Karski S., Witonska I. A., Kolesinska B., Kaminski Z. J. (2015). Fuel.

[cit44] Čuček L., Martín M., Grossmann I. E., Kravanja Z. (2011). Comput. Chem. Eng..

[cit45] Limayem A., Ricke S. C. (2012). Prog. Energy Combust. Sci..

[cit46] Aditiya H. B., Mahlia T. M. I., Chong W. T., Nur H., Sebayang A. H. (2016). Renewable Sustainable Energy Rev..

[cit47] Jambo S. A., Abdulla R., Mohd Azhar S. H., Marbawi H., Gansau J. A., Ravindra P. (2016). Renewable Sustainable Energy Rev..

[cit48] Zabed H., Sahu J. N., Suely A., Boyce A. N., Faruq G. (2017). Renewable Sustainable Energy Rev..

[cit49] Mohd Azhar S. H., Abdulla R., Jambo S. A., Marbawi H., Gansau J. A., Mohd Faik A. A., Rodrigues K. F. (2017). Biochem. Biophys. Rep..

[cit50] Ajit A., Sulaiman A. Z., Chisti Y. (2017). Food Bioprod. Process..

[cit51] Devarapalli M., Atiyeh H. K. (2015). Biofuel Res. J..

[cit52] Hofvendahl K., Hahn-Hägerdal B. (2000). Enzyme Microb. Technol..

[cit53] Berlowska J., Cieciura W., Borowski S., Dudkiewicz M., Binczarski M., Witonska I., Otlewska A., Kregiel D. (2016). Molecules.

[cit54] Binczarski M., Berlowska J., Stanishevsky A., Witonska I. (2016). RSC Adv..

[cit55] Ghantani V. C., Lomate S. T., Dongare M. K., Umbarkar S. B. (2013). Green Chem..

[cit56] Guo Z., Theng D. S., Tang K. Y., Zhang L., Huang L., Borgna A., Wang C. (2016). Phys. Chem. Chem. Phys..

[cit57] Södergård A., Stolt M. (2002). Prog. Polym. Sci..

[cit58] Nguyen C. M., Choi G. J., Choi Y. H., Jang K. S., Kim J.-C. (2013). Biochem. Eng. J..

[cit59] Tiwari K. P. , Pandey A. and Mishra N., Zentralblatt für Bakteriologie, Parasitenkunde, Infektionskrankheiten und Hygiene. Zweite Naturwissenschaftliche Abteilung: Mikrobiologie der Landwirtschaft, der Technologie und des Umweltschutzes, 1979, vol. 134, pp. 544–546 10.1016/s0323-6056(79)80078-6 549389

[cit60] Chaisu K., Charles A. L., Guu Y.-K., Yen T.-B., Chiu C.-H. (2014). APCBEE Proc..

[cit61] Concha Olmos J., Zúñiga Hansen M. E. (2012). Chem. Eng. J..

[cit62] Pietraszek P., Dybka K., Walczak P., Otlewska A., Rygała A., Ołtuszak-walczak E. (2014). Pol. J. Agron..

[cit63] Ghaffar T., Irshad M., Anwar Z., Aqil T., Zulifqar Z., Tariq A., Kamran M., Ehsan N., Mehmood S. (2014). J. Radiat. Res. Appl. Sci..

[cit64] Milcent S., Carrère H. (2001). Sep. Purif. Technol..

[cit65] Capon B. (1969). Chem. Rev..

[cit66] Cantor S. M., Peniston Q. P. (1940). J. Am. Chem. Soc..

[cit67] Barrows S. E., Storer J. W., Cramer C. J., French A. D., Truhlar D. G. (1998). J. Comput. Chem..

[cit68] Morpurgo S., Brahimi M., Bossa M., Morpurgo G. O. (2000). Phys. Chem. Chem. Phys..

[cit69] Silva A. M., da Silva E. C., da Silva C. O. (2006). Carbohydr. Res..

[cit70] de Lederkremer R. M., Marino C. (2003). Adv. Carbohydr. Chem. Biochem..

[cit71] Mirescu A., Prüße U. (2007). Appl. Catal., B.

[cit72] Odebunmi E. O., Ogunlaja A. S. (2011). Curr. Res. Chem..

[cit73] Campus T. (2013). Asian J. Chem..

[cit74] Wieland H. (1921). Berichte der deutschen chemischen Gesellschaft (A and B Series).

[cit75] Heyns K., Paulsen H. (1962). Adv. Carbohydr. Chem. Biochem..

[cit76] Heyns K., Paulsen H. (1957). Angew. Chem..

[cit77] Gogová Z., Hanika J. (2009). Chem. Eng. J..

[cit78] Delidovich I. V., Moroz B. L., Taran O. P., Gromov N. V., Pyrjaev P. A., Prosvirin I. P., Bukhtiyarov V. I., Parmon V. N. (2013). Chem. Eng. J..

[cit79] Megías-Sayago C., Ivanova S., López-Cartes C., Centeno M. A., Odriozola J. A. (2017). Catal. Today.

[cit80] Kuusisto J., Mikkola J. P., Sparv M., Wärnå J., Karhu H., Salmi T. (2008). Chem. Eng. J..

[cit81] Benkó T., Beck A., Frey K., Srankó D. F., Geszti O., Sáfrán G., Maróti B., Schay Z. (2014). Appl. Catal., A.

[cit82] Zhang H., Toshima N. (2013). J. Colloid Interface Sci..

[cit83] Witońska I., Frajtak M., Karski S. (2011). Appl. Catal., A.

[cit84] Karski S., Witońska I., Gołuchowska J. (2006). J. Mol. Catal. A Chem..

[cit85] Tathod A. P., Dhepe P. L. (2015). Bioresour. Technol..

[cit86] Hermans S., Devillers M. (2002). Appl. Catal., A.

[cit87] Witońska I. A., Walock M. J., Dziugan P., Karski S., Stanishevsky A. V. (2013). Appl. Surf. Sci..

[cit88] Armbrüster M., Schlögl R., Grin Y. (2014). Sci. Technol. Adv. Mater..

[cit89] Vlad-Cristea M. , Université Laval, 2007

[cit90] Sohst O., Tollens B. (1888). Justus Liebigs Ann. Chem..

[cit91] Kiely D. E. and Hash K. R., *US Pat*., 20080033205 A1, 2007

[cit92] Smith T. N., Hash K., Davey C.-L., Mills H., Williams H., Kiely D. E. (2012). Carbohydr. Res..

[cit93] Dijkgraaf P. J. M. , Oxidation of glucose to glucaric acid by Pt/C catalysts, PhD thesis, Technische Universiteit Eindhoven, 1989 10.6100/IR297031

[cit94] Merbouh N., Bobbitt J. M., Brückner C. (2002). J. Carbohydr. Chem..

[cit95] Timoshchuk V. A. (1995). Russ. Chem. Rev..

[cit96] Fox P. F. , Advanced dairy chemistry. Volume 3, Lactose, water, salts and vitamins, Springer Science+Business Media, 1997

[cit97] Okoro H. K., Odebunmi E. O. (2009). Int. J. Phys. Sci..

[cit98] Mehtiö T., Toivari M., Wiebe M. G., Harlin A., Penttilä M., Koivula A. (2015). Crit. Rev. Biotechnol..

[cit99] Green S. K. , Production of Renewable Fuels and Chemicals from Biomass-Dervied Furan Compounds, Doctoral Dissertations, University of Massachusetts, 2014, http://scholarworks.umass.edu/dissertations_2

[cit100] van Putten R.-J., van der Waal J. C., de Jong E., Rasrendra C. B., Heeres H. J., de Vries J. G. (2013). Chem. Rev..

[cit101] Lewkowski J. (2001). ARKIVOC.

[cit102] Kroh L. W. (1994). Food Chem..

[cit103] van Boekel M. A. J. S. (2001). Food.

[cit104] Martins S. I. F. S., Van Boekel M. A. J. S. (2005). Food Chem..

[cit105] Moreau C., Durand R., Razigade S., Duhamet J., Faugeras P., Rivalier P., Ros P., Avignon G. (1996). Appl. Catal., A.

[cit106] Hansen T. S., Woodley J. M., Riisager A. (2009). Carbohydr. Res..

[cit107] Witowski J., Jörres A. (2000). Peritoneal Dial. Int..

[cit108] Qi X., Watanabe M., Aida T. M., Smith Jr R. L. (2009). Green Chem..

[cit109] Wang J., Xu W., Ren J., Liu X., Lu G., Wang Y. (2011). Green Chem..

[cit110] Chheda J. N., Dumesic J. a. (2006). Science.

[cit111] Liu Y., Li Z., Yang Y., Hou Y., Wei Z. (2014). RSC Adv..

[cit112] Watanabe M., Aizawa Y., Iida T., Aida T. M., Levy C., Sue K., Inomata H. (2005). Carbohydr. Res..

[cit113] Yoshida T., Yanachi S., Matsumara Y. (2007). J. Jpn. Inst. Energy.

[cit114] Kuster B. F. M., van der Baan H. S. (1977). Carbohydr. Res..

[cit115] Wang S., Lin H., Chen J., Zhao Y., Ru B., Qiu K., Zhou J. (2015). RSC Adv..

[cit116] Seri K., Inoue Y., Ishida H. (2000). Chem. Lett..

[cit117] Lin H., Xiong Q., Zhao Y., Chen J., Wang S. (2017). AIChE J..

[cit118] Gomes F. N. D. C., Pereira L. R., Ribeiro N. F. P., Souza M. M. V. M. (2015). Braz. J. Chem. Eng..

[cit119] Hu S., Zhang Z., Song J., Zhou Y., Han B. (2009). Green Chem..

[cit120] Wu L., Song J., Zhang B., Zhou B., Zhou H., Fan H., Yang Y., Han B. (2014). Green Chem..

[cit121] Kuster B. F. M. (1990). Starch/Staerke.

[cit122] Wang T., Nolte M. W., Shanks B. H. (2014). Green Chem..

[cit123] Toftgaard Pedersen A., Ringborg R., Grotkjaer T., Pedersen S., Woodley J. M. (2015). Chem. Eng. J..

[cit124] Vigier K. D. O., Benguerba A., Barrault J., Jérôme F. (2012). Green Chem..

[cit125] Mimura N., Sato O., Shirai M., Yamaguchi A. (2017). ChemistrySelect.

[cit126] Shi N., Liu Q., Zhang Q., Wang T., Ma L. (2013). Green Chem..

[cit127] Perez Locas C., Yaylayan V. A. (2008). J. Agric. Food Chem..

[cit128] Carlini C., Giuttari M., Maria Raspolli Galletti A., Sbrana G., Armaroli T., Busca G. (1999). Appl. Catal., A.

[cit129] Benvenuti F., Carlini C., Patrono P., Raspolli Galletti A. M., Sbrana G., Massucci M. A., Galli P. (2000). Appl. Catal., A.

[cit130] Haworth W. N., Jones W. G. M., Stacey M., Wiggins L. F. (1944). J. Chem. Soc..

[cit131] Yu I. K. M., Tsang D. C. W. (2017). Bioresour. Technol..

[cit132] Mukherjee A., Dumont M. J., Raghavan V. (2015). Biomass and Bioenergy.

[cit133] Zhao H., Holladay J. E., Brown H., Zhang Z. C. (2007). Science.

[cit134] Hu S., Zhang Z., Zhou Y., Song J., Fan H., Han B. (2009). Green Chem..

[cit135] Qu Y., Li L., Wei Q., Huang C., Oleskowicz-Popiel P., Xu J. (2016). Sci. Rep..

[cit136] Zhang X., Zhang D., Sun Z., Xue L., Wang X., Jiang Z. (2016). Appl. Catal., B.

[cit137] Zhao S., Cheng M., Li J., Tian J., Wang X. (2011). Chem. Commun..

[cit138] Yu S.-B., Zang H.-J., Yang X.-L., Zhang M.-C., Xie R.-R., Yu P.-F. (2017). Chin. Chem. Lett..

[cit139] Reichstein T. (1926). Helv. Chim. Acta.

[cit140] van Bekkum H. , in Carbohydrates as Organic Raw Materials, ed. F. W. Lichtenthaler, VCH, Weinham, 1991

[cit141] Lew B. W. , *US Pat.*, 3326944, 1967

[cit142] Cottier L., Descotes G., Lewkowski J., Skowronski R. (1994). Pol. J. Chem..

[cit143] Chinaza Godswill A. (2017). Int. J. Adv. Acad. Res. Sci. Technol. Eng..

[cit144] Rafiqul I. S. M., Sakinah A. M. M. (2013). Food Rev. Int..

[cit145] Pappenberger G. and Hohmann H.-P., in Advances in biochemical engineering/biotechnology, 2013, vol. 143, pp. 143–188

[cit146] Na G. N., Kim S. A., Kwon O. C., Rhee M. S. (2015). J. Microbiol. Methods.

[cit147] Ansari A. R. M., Mulla S. J., Pramod G. J. (2015). Int. J. Adv. Pharm.

[cit148] Basch C. H., Kernan W. D. (2017). Global J. Health Sci..

[cit149] de Moraes Porto I. C., das Neves L. E., de Souza C. K., Parolia A., Barbosa dos Santos N. (2014). Oral Health Dent. Manag..

[cit150] Marques C. , Tarek R., Sara M. and Brar S. K., in Platform Chemical Biorefinery, 2016, pp. 217–227

[cit151] Ramírez-López C. A., Ochoa-Gómez J. R., Gil-Río S., Gómez-Jiménez-Aberasturi O., Torrecilla-Soria J. (2011). J. Chem. Technol. Biotechnol..

[cit152] Gustini L., Lavilla C., de Ilarduya A. M., Muñoz-Guerra S., Koning C. E. (2016). Biomacromolecules.

[cit153] Deshmukh A. G., Gogte B. B., Yenkie M. K. N. (2017). Int. J. Chem. Pharm. Anal..

[cit154] Pasupuleti S., Madras G. (2011). J. Appl. Polym. Sci..

[cit155] Nejand S. N. and Proulx C. R., *US Pat.*, 20170144124 A1, 2016

[cit156] Patel A. , *US Pat.*, 9271903 B2, 2014

[cit157] Wisnlak J., Simon R. (1979). Ind. Eng. Chem. Prod. Res. Dev..

[cit158] Anand A., Kulkarni R. D., Gite V. V. (2012). Prog. Org. Coatings.

[cit159] Geboers J., Van de Vyver S., Carpentier K., de Blochouse K., Jacobs P., Sels B. (2010). Chem. Commun..

[cit160] Negahdar L., Delidovich I., Palkovits R. (2016). Appl. Catal., B.

[cit161] Sasaki M., Kabyemela B., Malaluan R., Hirose S., Takeda N., Adschiri T., Arai K. (1998). J. Supercrit. Fluids.

[cit162] Teeri T. T. (1997). Trends Biotechnol..

[cit163] Romero A., Cantero D. A., Nieto-Márquez A., Martínez C., Alonso E., Cocero M. J. (2016). Green Chem..

[cit164] Huang Y. B., Fu Y. (2013). Green Chem..

[cit165] Muñoz-Guerra S. (2012). High Perform. Polym..

[cit166] Lavilla C., Alla A., Martínez De Ilarduya A., Benito E., García-Martín M. G., Galbis J. A., Mu-Noz-Guerra S. (2011). Biomacromolecules.

[cit167] Kiely D. E., Vishwanathan A., Jarman B. P., Manley-Harris M. (2009). J. Carbohydr. Chem..

[cit168] Henkensmeier D., Abele B. C., Candussio A., Thiem J. (2004). Macromol. Chem. Phys..

[cit169] Castellan A., Bart J. C. J., Cavallaro S. (1991). Catal. Today.

[cit170] U.S. Environmental Protection Agency OAQPS/TSD/EIB and N. 27711 Research Triangle Park , Background report AP-42 section 6.2. Adipic acid production, 1994

[cit171] Kruyer N. S., Peralta-Yahya P. (2017). Curr. Opin. Biotechnol..

[cit172] Deng Y., Mao Y. (2016). Biochem. Eng. J..

[cit173] Sousa A. F., Vilela C., Fonseca A. C., Matos M., Freire C. S. R., Gruter G.-J. M., Coelho J. F. J., Silvestre A. J. D., Carley A. F., Hutchings G. J. (2015). Polym. Chem..

[cit174] Bozell J. J., Petersen G. R. (2010). Green Chem..

[cit175] Werpy T. A. , Holladay J. E. and White J. F., Top Value Added Chemicals From Biomass: I. Results of Screening for Potential Candidates from Sugars and Synthesis Gas, Richland, WA, report, 2004

[cit176] de Jong E. , Dam M. A., Sipos L. and Gruter G.-J. M., in Biobased Monomers, Polymers, and Materials, American Chemical Society, 2012, pp. 1–13

[cit177] Wilsens C. H. R. M. , Exploring the application of 2,5-furandicarboxylic acid as a monomer in high performance polymers: Synthesis, characterization, and properties, PhD thesis, Technische Universiteit Eindhoven, 2015 10.6100/IR783770

[cit178] Wang F., Zhang Z. (2017). J. Taiwan Inst. Chem. Eng..

[cit179] Lolli A., Amadori R., Lucarelli C., Cutrufello M. G., Rombi E., Cavani F., Albonetti S. (2016). Microporous Mesoporous Mater..

[cit180] Lolli A., Albonetti S., Utili L., Amadori R., Ospitali F., Lucarelli C., Cavani F. (2015). Appl. Catal., A.

[cit181] Ait Rass H., Essayem N., Besson M. (2015). ChemSusChem.

[cit182] Zuo X., Venkitasubramanian P., Busch D. H., Subramaniam B. (2016). ACS Sustainable Chem. Eng..

[cit183] Han X., Li C., Liu X., Xia Q., Wang Y. (2017). Green Chem..

[cit184] Carlos Morales-Huerta J., Martínez De Ilarduya A., Muñoz-Guerra S. (2016). Polymer.

[cit185] Gandini A., Coelho D., Gomes M., Reis B., Silvestre A. (2009). J. Mater. Chem..

[cit186] Carlmark A., Larsson E., Malmström E. (2012). Eur. Polym. J..

[cit187] Adcock L. H., Gray C. H. (1956). Nature.

[cit188] Sestoft L. (1985). Acta Anaesthesiol. Scand., Suppl..

[cit189] Bruggeman J. P., de Bruin B.-J., Bettinger C. J., Langer R. (2008). Biomaterials.

[cit190] Engels H. W., Pirkl H. G., Albers R., Albach R. W., Krause J., Hoffmann A., Casselmann H., Dormish J. (2013). Angew. Chem., Int. Ed..

[cit191] Avar G. , Meier-Westhues U., Casselmann H. and Achten D., in Polymer Science: A Comprehensive Reference, 2012, pp. 411–441

[cit192] Gagro D. (2010). Eur. Coat. J..

[cit193] Henkensmeier D., Abele B. C., Candussio A., Thiem J. (2004). Polymer.

[cit194] Matsuo T. , Kamikawa M., Kondo T. and Maeda N., *US Pat.*, 20140024793 A1, 2014

[cit195] Gandini A., Silvestre A. J. D., Neto C. P., Sousa A. F., Gomes M. (2009). J. Polym. Sci. Part A Polym. Chem..

[cit196] Gomes M., Gandini A., Silvestre A. J. D., Reis B. (2011). J. Polym. Sci. Part A Polym. Chem..

[cit197] Sipos L. , *US Pat.*, 20110282020 A1, 2011

[cit198] Pan T., Deng J., Xu Q., Zuo Y., Guo Q.-X., Fu Y. (2013). ChemSusChem.

[cit199] Kizuka K., Inoue S.-I. (2015). Open J. Org. Polym. Mater..

[cit200] Wang L., Zhao B., Liu B., Yu B., Ma C., Su F., Hua D., Li Q., Ma Y., Xu P. (2010). Bioresour. Technol..

[cit201] Dumbrepatil A., Adsul M., Chaudhari S., Khire J., Gokhale D. (2008). Appl. Environ. Microbiol..

[cit202] Göksungur Y., Güvenç U. (1997). J. Chem. Technol. Biotechnol..

[cit203] Wee Y. J., Kim J. N., Yun J. S., Ryu H. W. (2004). Enzyme Microb. Technol..

[cit204] Modelska M., Berlowska J., Kregiel D., Cieciura W., Antolak H., Tomaszewska J., Binczarski M., Szubiakiewicz E., A Witonska I. (2017). Molecules.

[cit205] Rogowski J., Andrzejczuk M., Berlowska J., Binczarski M., Kregiel D., Kubiak A., Modelska M., Szubiakiewicz E., Stanishevsky A., Tomaszewska J., Witonska I. A. (2017). Molecules.

[cit206] Berlowska J., Pielech-Przybylska K., Balcerek M., Cieciura W., Borowski S., Kregiel D. (2017). Energies.

[cit207] Patelski P., Berłowska J., Dziugan P., Pielech-Przybylska K., Balcerek M., Dziekońska U., Kalinowska H. (2015). J. Food Eng..

